# Combination of tunicamycin with anticancer drugs synergistically enhances their toxicity in multidrug-resistant human ovarian cystadenocarcinoma cells

**DOI:** 10.1186/1475-2867-7-5

**Published:** 2007-04-18

**Authors:** Donavon C Hiss, Gary A Gabriels, Peter I Folb

**Affiliations:** 1 Division of Clinical Pharmacology, Department of Medicine, University of Cape Town, Observatory, 7925, South Africa; 2 Department of Medical BioSciences, University of the Western Cape, 7535, Bellville, South Africa; 3 Medical Research Council, 7505, Tygerberg, South Africa

## Abstract

**Background:**

The pharmacologic modulatory effects of the antibiotic, tunicamycin (TM), on multidrug-resistant human UWOV2 ovarian cancer cells are reported. The UWOV2 cell line was derived from a cystadenocarcinoma in a patient refractory to combination chemotherapy with actinomycin D, vincristine (VCR), cis-diaminedichloroplatinum (II) (CDDP) and doxorubicin (DXR). In an attempt to explain drug resistance in this cell line, we examined the effects of TM on their sensitivity to various anticancer drugs, the uptake, efflux and retention of [^3^H]VCR, and their ability to bind [^14^C]DXR and [^3^H]azidopine (AZD), a photoaffinity label of the multidrug transporter, P-glycoprotein (Pgp).

**Results:**

TM effectively decreased the EC_50 _for DXR, EXR, VCR and CDDP, thus enhancing their cytotoxicity. The antibiotic also prolonged the intracellular retention time of [^3^H]VCR and increased the binding of both [^14^C]DXR and [^3^H]AZD to the cells.

**Conclusion:**

It is concluded that the pharmacomodulatory effects of TM in these cells are mediated by global inhibition of protein and glycoprotein synthesis and synergistic interaction with antineoplastic drugs. The ability of TM to enhance the sensitivity of drug resistant tumour cells may have impact on the design and optimization of novel resistance modifiers to improve the efficacy of combination treatment of intractable neoplasms.

## Background

The role of post-translational modification of proteins, such as N-glycosylation, in normal and transformed processes is well documented [1-9]. This knowledge has prompted explicit pharmacological interest in compounds that can interfere with glycoprotein processing at the cellular level [1,3-19]. The nucleoside antibiotic, tunicamycin (TM), is a prototype of substances that exert potent inhibitory effects on protein maturation [1,2,4,20-24]. TM has been applied, *in vitro*, primarily to discern the functional significance of N-glycosylation in living systems, including cell proliferation and survival [25-28], drug sensitivity and resistance of tumour cells to antineoplastic drugs [12,25,28-30], and programmed cell death or apoptosis [21,23,27,28,31-41].

Programmed cell death is mediated through several mechanisms, including the endoplasmic reticulum (ER) stress or unfolded protein response (UPR) [35,38,41-53]. The ER stress response typically involves transcription factor CHOP/GADD153 (growth arrest and DNA damage 153) and death receptor 5 (DR5) [21,23,51-54]. Perturbation of N-glycosylation in the ER results in the expression of aberrant or misfolded proteins which, in turn, activate the UPR to orchestrate their decomposition and disposal by the ER-associated protein degradation (ERAD) machinery [55-57]. The importance of the UPR in oncogenesis and resistance of cancers to chemotherapeutic drugs is increasingly being acknowledged [19,35]. A notable corollary in this regard is the finding that bortezomib [19,21], a potent and selective inhibitor of the ubiquitin-proteasome system (UPS, which likewise serves to identify and remove malformed proteins [19,45,52], is also proapoptotic – an effect triggered by TM and thapsigargin (classic ER stress inducers) via a c-Jun-terminal kinase (JNK)-dependent mechanism [21].

The multifactorial basis and complex nature of the molecular interactions in diseases with a high prevalence [58-60] also underscore the difficulties in predicting ovarian tumour chemoresponsiveness and curative potential [60,61]. The poor prognosis of ovarian carcinoma is often ascribed to the development of multidrug resistance (MDR) [62,63]. This lack of response to chemotherapy is observed in many tumour types [64] and its circumvention is the subject of keen research [65]. Many chemical agents, referred to as biological response or resistance modifiers have been demonstrated to alter chemosensitivity in refractory tumour cells and are potentially useful in clinical cancer therapeutics [66,67]. Recently, several efforts have been made to suppress N-glycosylation, using TM, as a molecular tenet to overcome experimental drug resistance [25,28-30,68]. The rationale behind this approach is based on the assumption that inhibition of the processing and maturation of P-glycoprotein (Pgp), an ATP-dependent efflux pump that prevents intracellular accumulation of cytotoxic antineoplastic drugs in tumour cells, will necessarily alter its structural-functional integrity and mediation of MDR [12,14,25,69-72].

Furthermore, there has been a renewed focus and increased perspective on multicomponent therapeutics for the innovation of drug discovery towards pharmacological intervention with several compounds that interact with diverse targets [59], especially with regard to combination response reference models [58,73,74]. In this study, we examined the pharmacomodulatory effects of TM in the context of its interaction with the anticancer drugs doxorubicin (DXR), epirubicin (EXR), vincristine (VCR) and cisplatin (cis-diamine-dichloroplatinum [II], CDDP), and the possible mechanistic relation of such combinations to the expression of drug resistance in human UWOV2 ovarian cancer cells. The UWOV2 cell line was derived from a cystadenocarcinoma in a patient refractory to combination chemotherapy with actinomycin D, VCR, CDDP and DXR. Accordingly, this cell line was taken to represent a cancer phenotype consistent with *in-vivo*-acquired and/or intrinsic MDR relevant to determining the chemotherapeutic promise of modulators used in combination with anticancer agents.

## Methods

### Radioisotopes, drugs and chemicals

Doxorubicin (adriamycin) and epirubicin (epidoxorubicin) (Farmitalia Carlo Erba, Milan, Italy), cis-diaminedichloroplatinum (II) (Lennon, South Africa), gentamicin sulphate (Roussel Laboratories, South Africa), vincristine sulphate, 3- [4,5-dimethylthiazole-2-yl]-2,5-diphenyltetrazolium bromide (MTT) (Sigma Chemical Co., St. Louis, MO, USA), trypsin 1:250 (Difco Laboratories, Detroit, MI, USA), PBS Dulbecco 'A' (Oxoid, UK), RPMI-1640 and McCoy's 5A culture medium (Gibco, UK), tunicamycin, penicillin G (Boehringer Mannheim, Germany), EDTA (Merck Chemicals, Germany), radioactive isotope precursors and drugs (Amersham Biosciences, UK), Bio-Rad protein assay dye reagent concentrate (Bio-Rad Laboratories, UK) were used in this study. All other reagents were of the highest analytical grade and were obtained from either Sigma Chemical Co. or Merck Chemicals. P-glycoMab (consisting of lyophilized C219 monoclonal antibody and isotype-matched negative antibody, biotinylated anti-mouse antibody, avidin and biotinylated horseradish peroxidase visualization system) was purchased from Centocor Diagnostics, Tongeren, Belgium.

### Cell culture of UWOV2 cells

The UWOV2 ovarian carcinoma cell line, derived from a cystadenocarcinoma in a patient who had not responded to combination chemotherapy with actinomycin D, vincristine, cisplatin and doxorubicin [75], was maintained in RPMI-1640 medium supplemented with 5% heat-inactivated foetal calf serum (HIFCS), penicillin G (100 U/ml) and streptomycin sulphate (100 μg/ml) or gentamicin sulphate (50 μg/ml) at 37°C in 5% CO_2_:air and 85% relative humidity. The expression of Pgp in UWOV2 cells was demonstrated previously using the monoclonal antibody, C219, and the avidin-biotin-immunoperoxidase P-glycoCHEK diagnostic kit with the drug-sensitive human acute lymphoblastic leukemia cell line, CCRF-CEM (Pgp-negative) and its drug-resistant derivative, CEM-VLB100 (strongly Pgp-positive) as controls [76]. The BG-1 ovarian carcinoma cell line and its adriamycin-resistant derivative, BG-1/ADR were a gift from Dr C.A. Wallen (Bowman Gray School of Medicine, North Carolina, USA) and grown in McCoy's 5A medium containing 10% HIFCS, 100 U/ml insulin, 200 mM L-glutamine and antibiotics. Cells were routinely subcultured with trypsin-EDTA (0.25%–0.02%, w/v) in Ca^+2^- and Mg^+2^-free PBS and maintained in the logarithmic phase of growth. Cells were periodically tested and found to be free of mycoplasma contamination.

### Precursor-incorporation-inhibition studies

The effects of TM on the incorporation of radiolabelled precursors [^35^S]methionine (>1000 Ci/mmole) and [^3^H]mannose (30–60 Ci/mmole) or [^3^H]glucosamine (20–40 Ci/mmole) into trichloroacetic acid-insoluble macromolecules (proteins and glycoproteins) were measured in UWOV2 cells as described previously [29].

### In vitro cytotoxicity assays

To determine the effect of TM on the cytotoxicity of DXR, EXR, VCR and CDDP, preconfluent cells from stock cultures were detached with trypsin-EDTA (0.25%–0.02%, w/v) in PBS, washed twice with PBS and resuspended in complete culture medium to obtain single-cell suspensions. Standardization of cell numbers in individual wells of a 96-well flat-bottom microtiter plate was achieved by a linear correlation (r = 0.98) between cell number and absorbance up to a maximum density of 3.5–4.5 × 10^4 ^cells/well. Cells were seeded at a density of 3 × 10^3 ^cells/well in a total volume of 200 μl as follows: After trypsinization, cells were rinsed twice with PBS, resuspended in 10 ml complete culture medium and repeatedly pipetted to ensure a homogeneous mixture during dispensing of 100-μl aliquot/well. The cells were then allowed to attach and grow for 48–72h. Cytotoxic drugs were dissolved in PBS and sterilized through 0.22-μm disposable filters (Millipore, Millex-GV). The drugs were diluted in culture medium free of phenol red (RPMI-1640-selectamine kit) to avoid interference with spectrophotometric assays. Tunicamycin stock solutions were prepared by dissolving the contents of a 10-mg vial in 25 mM NaOH and then diluting to 0.8 mg/ml TM and 10 mM NaOH with pyrogen-free distilled-deionized water. The solution was sterilized by passing through a 0.22-μm disposable filter and diluted to final concentrations in culture medium. After the addition of drugs and TM to octuplicate wells, cells were incubated for a further 72h. The cytotoxicity of drug in the TM-treated (combination) and TM-free cultures (control) was determined by the MTT (3–4,5-dimethylthiazol-2,5-diphenyltetrazolium bromide) assay [77]. In this assay, 20 μl of MTT (5 mg/ml in sterile PBS) were added to each well and the plates incubated for 5h at 37°C. Plates were then centrifuged at 400 × g for 5 min to pellet any floating cell aggregates. The supernatant was aspirated and the formazan crystals dissolved in DMSO (200 μl/well). Absorbances were read by a microplate reader (Titertek Multiskan model MCC/340) at a sample wavelength of 540 nm and a reference wavelength of 630 nm. The EC_50 _for TM, DXR, EXR, VCR and CDDP were determined by non-linear regression of sigmoidal dose-response curves using Graphpad Prism (Version 4.03, GraphPad Software, San Diego California USA, ). The best-fit EC_50 _(concentration that produces 50% of the maximal response) values for each drug alone or in combination with a fixed concentration of 5 μg/ml TM were subjected to statistical evaluation as described in "Data analysis".

### Assay of [G-^3^H]vincristine sulfate uptake and efflux

To assay VCR uptake and efflux, cells were seeded at a density of 5 × 10^4 ^cells/ml in 24-well plates and allowed to grow for 48h under standard conditions. Cells were pretreated with 5 μg/ml TM for 16h to suppress *de novo* protein and glycoprotein synthesis. Parallel controls were set up. Total cellular accumulation of VCR was determined by exposing cells in quadruplicate wells to [G-^3^H]VCR sulphate (30 nM or specific activity 9.48 cpm/pmol) in the continued absence or presence of 5 μg/ml TM in a final volume of 0.5 ml for various incubation times. At the end of each incubation period, cells were washed three times with 1 ml ice-cold PBS and solubilized in 0.5 ml of 1% SDS/0.3 M NaOH. One aliquot (0.4 ml) was neutralized by the addition of 0.2 ml of 2 M acetic acid and mixed with 10 ml scintillation fluid (Beckman Ready-Solv EP) and counted in a Beckman scintillation spectrometer. Intracellular drug at each time point was determined by subtracting the value for non-specific/surface-bound drug obtained by incubation with 100 μM unlabelled VCR for 30s at 4°C from the value for total drug. The other aliquot (0.1 ml) was assayed for total cellular protein. Vincristine efflux was measured by loading control and TM-pretreated cells with [^3^H]VCR for 60 min (0-time value for efflux) followed by washing preloaded cells three times with ice-cold PBS and subsequently incubating at 37°C in serum- and antibiotic-free medium (2 ml) for various time intervals. The absence or presence of TM was maintained throughout the post-incubation periods. Cells were harvested as described for uptake studies. A large volume ratio (i.e., preloading volume/post-incubation volume of 4) was ensured during efflux and retention measurements to minimize reutilization of extruded drug.

### Binding of [^3^H]azidopine and [^14^C]doxorubicin to UWOV2 cells

The specific binding of [^3^H]AZD (46 Ci/mmol) and [^14^C]DXR (50–62 mCi/mmol) to UWOV2 cells in the absence (control) or presence of TM (TM-treated) was measured by a modification of the procedure described earlier [78]. Cells were seeded at a density of 5 × 10^4 ^cells/ml in 24-well plates. Confluent cell monolayers were cooled by placing the plates on ice for 10 min, washed four times with 1 ml cold PBS, pH 7.4 (to remove culture medium serum glycoproteins) and maintained for 60 min at 4°C with binding buffer (10 mM glucose, 3 mM ATP and 5 mM MgCl_2_ in 10 mM Tris-HCl, pH 7.4) containing [^3^H]AZD or [^14^C]DXR in serial dilutions of 10–80 nM. Following this incubation period, the cells were washed five times with cold PBS to remove unbound [^3^H]AZD and [^14^C]DXR. Cells were then solubilized in 0.1 M NaOH and samples were counted to determine the amount of AZD or DXR bound to the cells, and aliquots were removed for protein determination. Specific binding was distinguished from non-specific binding to cells and plastic wells by dilution with excess (100 μM) unlabelled drug. To examine competitive binding between DXR and AZD, cells were exposed to equimolar concentrations of both compounds (unlabelled DXR and labelled AZD) and the specific binding of AZD determined as described above. The specific activity of the UWOV2 cell surfaces/receptors (B_max_) and the binding affinity constant (K_d_) for DXR and AZD were determined by non-linear regression (one-site binding hyperbola) of specific binding data, using GraphPad Prism .

### Protein determination

The total cellular protein content was estimated by utilizing the Bio-Rad protein dye-binding assay kit.

### Data analysis

Statistical analysis was performed on the variables in this study using either the Student's two-tailed t-test or one-way ANOVA. The level of significance was set at p < 0.05. Values are representative of the means ± SEM of 3 experiments (n = 8 for each experiment), unless indicated otherwise. Actual p values are presented. An experimental design for comparison of a single anticancer drug dose-response relation with that of the same anticancer drug in combination with a fixed concentration of 5 μg/ml TM was used and the measured responses compared to both the Loewe additivity and Bliss independence reference models of synergy [73,79,80] using the CombiTool computer programme (version 2.001, IMB Jena Biocomputing Group, ). The drug interaction index (I_x_) was calculated according to the method of Chou and Talalay [81] using CombiTool. I_x _values are geometric means ± SEM (standard error of the mean) of multiple effect levels (fraction affected, F_a_) 0.1, 0.2, 0.3,..., 0.99 (i.e., EC_10_, EC_20_, EC_30_,..., EC_99_) for each drug in the dose range 10^-4 ^to 10^1 ^μg/ml in the presence of a fixed concentration of 5 μg/ml TM: I_x _< 1 ⇒ synergy; I_x _= 1 ⇒ additivity and I_x_>1 ⇒ antagonism. The Loewe dose additivity model is defined by the equation dx/Dx + dy/Dy = 1, where Dx and Dy represent the concentrations of individual drugs required to exert the same effect as concentrations dx and dy used in combination. In this case, dx would be the effective inhibitory concentration (EC) of the drug used in combination with TM and Dx the EC of the drug alone. Likewise, dy would be the fixed concentration of TM used in combination with the drug and Dy the EC of TM (determined from the individual dose-response relation for TM) to produce the same effect. Student t-tests were performed to evaluate significant differences in I_x _compared to a null hypothesized I_x _value of 1. The Bliss independence model is defined by the equation: E_xy _= E_x _+ E_y _- E_x_E_y _for 0 < E < 1 and where E_xy _is the additive effect of drugs x and y as predicted by their individual effects E_x _and E_y_. In this case, E_xy _would be the effect (fractional survival) of the drug used in combination with TM, and E_x _and E_y _the fractional survival of cells exposed to the drug alone and to the drug in combination with TM, respectively. EC_50 _data are best-fit values obtained from non-linear regression analysis of the sigmoidal dose-response relation for each drug alone or in combination with TM. The potency ratio and associated 95% CI (confidence interval) were computed according to the method of Fieller [82] by subtracting the log EC_50 _of drug in combination with TM from the log EC_50 _of drug alone and back-transformation (antilogarithm) of data. GraphPad QuickCalcs, Graphpad Prism (Version 4.03, GraphPad Software, San Diego California USA, ), SigmaPlot (version 9.01) and SigmaStat (Version 3.11) (Systat Software, Inc. 501 Canal Blvd, Suite E, Point Richmond, CA 94804-2028, USA, ) were used for data and graphic analysis.

## Results

### Precursor-incorporation-inhibition studies

The inhibitory effects of TM on UWOV2 cells were measured by following the incorporation of radiolabelled precursors into proteins and glycoproteins. In the presence of TM, a marked inhibition of protein (Figure 1A) [see Additional file 1] and glycoprotein (Figure 1B) [see Additional file 1] synthesis was observed in UWOV2 cells. The incorporation of [^35^S]methionine into cellular protein was greatly diminished at all incubation times, except at 4h (Figure 1A) [see Additional file 1]. The concentration-dependent effect of TM on [^3^H]glucosamine incorporation by UWOV2 cells is shown in Figure 1B [see Additional file 1]. At concentrations less than 0.05 μg/ml, TM had no inhibitory activity, but at higher concentrations (0.5–50 μg/ml) the antibiotic significantly impaired glycoprotein synthesis compared to control. The inhibitory effect of TM was verified by similar analysis of the incorporation of [^3^H]mannose into glycoprotein in the absence (control) or presence of 5 μg/ml TM (TM-treated) at different time intervals following an initial 16h pre-incubation with the antibiotic (Figure 2) [see Additional file 2].

### In vitro cytotoxicity assays

The effects of TM on the viability of UWOV2 cells are depicted in Figure 3 [see Additional file 3]. TM did not affect cell viability in the concentration range 0.0001–5 μg/ml and survival was consistently greater than 95% or similar to control (i.e., cells not treated with TM). The best-fit estimate of the EC_50 _for TM in UWOV2 cells was 23.6 μg/ml (95% CI: 10.34 to 53.96), compared to a reference ovarian cancer cell line BG-1 (EC_50_, TM = 16.81 μg/ml; 95% CI: 5.87 to 48.17) and its adriamycin (DXR)-resistant variant, BG-1/ADR (EC_50_, TM = 64.84 μg/ml; 95% CI: 18.98 to 221) (survival curves of TM for BG-1 and BG-1/ADR are not shown). Dose-response curves for UWOV2 cells treated with different drugs alone or in combination with 5 μg/ml TM are presented in Figure 3 [see Additional file 3] and the results obtained from non-linear regression of the sigmoidal dose-response relation for each drug alone or in combination with TM are summarized in Table 1 [see Additional file 4]. The cells displayed a high degree of resistance to VCR (EC_50 _= 23.2 μg/ml; 95% CI: 9.11 to 59.07) relative to the other drugs: 5.86-fold > DXR (95% CI: 2.46 to 14), 8-fold > EXR (95% CI: 1.86 to 35.17) and 5.67-fold > CDDP (95% CI: 2.43 to 13.35). One-way ANOVA revealed no major variations in the log EC_50 _of DXR and EXR (p = 0.711), DXR and CDDP (p = 0.971) as well as EXR and CDDP (p = 0.684), but significant differences between the log EC_50 _of DXR and VCR (p = 0.041), EXR and VCR (p = 0.016), and CDDP and VCR (p = 0.044). TM potentiated the cytotoxicity of all the drugs by effectively decreasing their EC_50 _and, correspondingly, increasing the potency ratio for each drug in the following order of magnitude: EXR (102-fold) > DXR (88-fold) > CDDP (17-fold) > VCR (5-fold) (Table 1). Unpaired t-tests for the differences in log EC_50 _for drug alone and drug in combination with TM yielded p values less than 0.001 for DXR, EXR and CDDP, but not for VCR (p = 0.335). Thus, although TM increased the potency ratio for VCR in UWOV2 cells, the enhanced toxicity was not statistically significant.

### Drug synergy analysis

The influence of TM on the efficacy of the anticancer drugs under study was further appraised in respect to the differences of the measured responses and the expected values generated from the CombiTool programme for the Loewe additivity and Bliss independence models for drug interaction (Figure 4) [see Additional file 5]. Loewe and Bliss antagonism were observed at the lower range (lower effect levels) of drug concentrations (0.0001 to 0.003 μg/ml) for DXR and CDDP and at 0.0001 for VCR (not shown), whereas all the drugs in combination with TM displayed Loewe and Bliss synergism at higher effect levels (Figures 4A and 4B, respectively) [see Additional file 5]. The synergistic action between TM and the different anticancer drugs was confirmed by the generalized isobolar median-effect method, using the CombiTool computer programme. The results, presented in Figure 4C [see Additional file 5], show that synergism occurs at effect levels (fraction affected, F_a_) of 0.2 and above. The combination index (I_x_) for each drug at multiple effect levels was also compared with a null hypothesized value of 1 and yielded p values < 0.001 in all cases, thus indicating a high degree of synergism, according to the Loewe additivity model, for all the TM-drug combinations (Table 1).

### Assay of vincristine uptake and efflux

Based on the relatively high degree of resistance of UWOV2 cells to VCR and the synergistic action of TM on its potency, an investigation into the direct effects which TM might exert on the transport of VCR in these cells was undertaken. This, along with the assumption that VCR transport in these cells is coupled to the expression of Pgp, therefore provided an approach that could reflect a general association between inhibition of protein and glycoprotein synthesis and the drug transport mechanism. The effects of TM on the transport of [G-^3^H]VCR into (uptake) and out of (efflux) UWOV2 cells are shown in Figure 5 [see Additional file 6]. TM had no appreciable effect on the saturable uptake of VCR by these cells (Figure 5A) [see Additional file 6], but VCR efflux was significantly and consistently inhibited at post-incubation times 60 min (p < 0.001), 120 min (p = 0.004) and 180 min (p = 0.004) (Figure 5B) [see Additional file 6]. Data obtained for separate experiments which emulated efflux studies, on the retention of VCR in response to TM treatment, are summarized in Figure 6 [see Additional file 7]. TM induced a consistent 20% increase in the fractional retention of VCR in UWOV2 cells at post-incubation times 60 min (p = 0.004), 80 min (p = 0.029) and 100 min (p = 0.027). The results show that by lowering the efflux rate of VCR from the cells, probably via inhibition of Pgp synthesis and function, TM concomitantly raises the amount of drug retained in the cells. Such increased retention of VCR brought about by TM may well explain the observed increased efficacy or potency of VCR and the other drugs when combined with TM, especially in view of the molecular components targeted in the experimental setup. Therefore, overall inhibition of both protein and glycoprotein synthesis as well as drug efflux which possibly involve Pgp in the interconnected system may account for the observed enhancing effects of TM on drug cytotoxicity in UWOV2 cells.

### Binding of azidopine and doxorubicin to UWOV2 cells

The MDR status of UWOV2 ovarian carcinoma cells was demonstrated, in part, by measuring the specific binding of [^3^H]AZD, a photoactive dihydropyridine calcium channel blocker known to bind to Pgp, and [^14^C]DXR to intact cells in culture at 4°C in the absence (control) or presence of 5 μg/ml TM (TM-treated) following an initial 16h pre-incubation at 37°C with or without the antibiotic. The binding of DXR to the cells was saturable in the linear concentration range of 0–80 nM (Figure 7A) [see Additional file 8]. The measured binding affinity for DXR in control cultures was K_d _= 28.48 ± 14.94 nM (95% CI: 0–59.5) and the receptor/cell surface specific activity was B_max _= 0.61 ± 0.14 pmol/mg (95 % CI: 0.33 to 0.89). In the presence of TM, the B_max _for DXR was increased 2.87-fold (95 % CI: 1.05 to 7.33, p = 0.045) as determined by Fieller's ratio of means test. The unpaired t-test for the difference in K_d _for control and that for TM-treated cells (K_d _= 60.82 ± 26.66 nM (95 % CI: 5.52 to 116) indicated no significant change in this parameter for DXR (p = 0.331). Taken together, the results show that the increased binding of DXR to UWOV2 cells in the presence of TM is probably caused by a mechanism which facilitates DXR attachment to the cells. This supposition was corroborated by an analysis of the results in terms of fractional occupancy of binding sites as predicted by the law of mass action at equilibrium (saturation), i.e., a plot of fractional occupancy ([DXR]/([DXR] + K_d_) vs [DXR]/K_d _yielded a rectangular hyperbola (not shown) similar to Figure 7A [see Additional file 8]. The plot revealed that maximal occupancy of DXR binding sites was attained at much lower concentrations of DXR when cells had been treated with TM. Although the precise molecular mechanism has yet to be established, it is apparent that TM pre-treatment facilitates DXR binding on UWOV2 cell surfaces. The binding of the photoaffinity label of Pgp, [^3^H]AZD, to intact cells (control) and cells treated with TM was studied as described for DXR. Binding of AZD remained linear (i.e., non-saturation binding) between 0 and 80 nM (Figure 7B) [see Additional file 8]. TM treatment also significantly increased the binding of AZD to cells at all the concentrations studied (p = 0.008). In the presence of equimolar concentrations (range 30–80 nM) of unlabelled DXR, the binding of labelled AZD to UWOV2 cells was significantly reduced (Figure 7B, p < 0.005) [see Additional file 8], suggesting interaction of DXR with Pgp. This reduction of AZD binding to UWOV2 cells in the presence of DXR was attenuated by treatment of cells with TM (Figure 7B, p < 0.005) [see Additional file 8]. Non-linear regression analysis of AZD binding did not converge and could not be performed as the incubation was in the linear region, i.e., saturation (equilibrium) was not reached. This could well have been accomplished in a study design with log increments of AZD concentration, but was not considered crucial for the purpose of this investigation.

## Discussion

Effective management of ovarian carcinomas is often limited by their relative lack of response to chemotherapy. Ovarian tumours have been reported to manifest the MDR phenotype variously [61-63,70,83]. In addition, the development of resistance to drugs that are most active against ovarian cancer may occur through mechanisms other than the expression of Pgp [84,85]. Despite this and the large variability in Pgp levels observed in ovarian cancer [70,83,86], the insensitivity of this malignancy to chemotherapy can be correlated with the expression of the *mdr*1 gene product [61,87-89]. We have previously demonstrated the expression of Pgp in UWOV2 cells using the P-glycoCHEK immunocytochemical diagnostic kit and verified its presence in these cells with SDS-PAGE of purified plasma membrane samples immunoprecipitated with Pgp-specific mAbs MRK-16 and C219 [76]. Ovarian carcinoma cell lines, such as UWOV2, derived from clinically drug-resistant patients, are useful for *in vitro *anticancer drug screening and the identification of valuable novel treatment regimens [88,90].

In our system, the inhibition of *de novo *synthesis of proteins and glycoproteins can be explained by the potent suppression by TM of [^35^S]methionine, [^3^H]mannose and [^3^H]glucosamine incorporation into UWOV2 cells. A model for such TM-mediated translational inhibition (i.e., attenuation of the transfer of oligosaccharide units to specific asparaginyl (Asn) residues in nascent polypeptides within the lumen of the ER to form N-linked glycoproteins) illustrates why an inadequate supply of the lipid-linked oligosaccharide Glc3Man9GlcNAc2-P-P-dolichol precursor may block the maturation of glycoproteins and their translocation to membranes [91]. Agents that perturb N-glycosylation and protein folding in the ER induce the UPR response and eventually growth arrest or apoptosis if the homeostatic compensatory mechanisms are insufficient to cope with unabated ER stress [42,51,52].

Cellular insults by inducers of ER stress such as TM and thapsigargin, activate the ubiquitin-proteasome system (UPS), a catalytic proteinase complex which neutralizes proteins with abnormal conformations [19,45,52]. Treatment of MDR cells with an inhibitor of N-glycosylation increases ubiquitinylation of Pgp [72]. Ubiquitinylation is a recognized signal for the upregulation of protein degradation and a process that would affect the stability and function of Pgp. Inhibition of N-linked glycosylation by TM has also been associated with enhanced turnover of misfolded proteins which is carried out by the UPS [41,92]. The precursor form of Pgp is non-glycosylated with apparent molecular mass (MW_app_) varying between 120 to 140 kDa [12]. Also, reduction in the MW of Pgp from 180 kDa to 150 kDa, upon exposure of cells to TM, is well established [68]. Correspondingly, Pgp has been reported to be differentially glycosylated resulting in heterogeneous forms of MDR-associated glycoproteins in different cell lines which may or may not correlate with drug resistance [93].

Since N-linked glycosylation occurs in the ER, TM as an inducer of the UPR may affect the proper folding of Pgp and prevent its insertion into the plasma membrane thus altering membrane topology and permeability [68]. However, a recent report asserted that Pgp-mediated vinblastine efflux in rat hepatoma cells was increased despite impaired glycosylation and induction of the ER stress response by TM, 2-deoxyglucose and thapsigargin [69]. Thapsigargin and TM, agents that induce ER stress, have been shown to sensitize breast (MCF-7) and prostate (DU145) cancer cells to the cytotoxic effects of diindolylmethane (DIM), raising the possibility that stressed cells also have increased sensitivity to cytotoxic agents [23]. Evidence in support of such a notion has been provided by a study in which the combined exposure of myeloma cells to the proteasome inhibitor MG-132 and the ER stressor TM resulted in a synergistic cytocidal effect [94]. Similarly, bortezomib – another proteasome inhibitor with substantial antitumour efficacy [19,21] – markedly improved the sensitivity of pancreatic cancer cells to cisplatin and further promoted apoptosis induced by TM and thapsigargin [21]. Moreover, the establishment of a functional link between N-glycosylation and apoptosis [31,40,41] and the observation that CDDP-resistance in an ovarian carcinoma is coupled with a defect in programmed cell death [95] is interesting. Therefore, delineation of the tangible effects of TM on the ER signalling pathway in relation to tumour cell death may offer prospects for rational anticancer drug design and more effective chemotherapeutic combinations [20,21,23,48,54,69,96].

The increased binding of DXR and AZD, in particular, to intact UWOV2 cells in the presence of TM implies that the antibiotic may exert its effects by modulating cell surface binding activity either through direct membrane perturbations and/or alterations in the integrity of Pgp [97]. Such effects may ultimately be responsible for changes in Pgp-substrate, -ligand and -inhibitor absorption, distribution and excretion [98]. Structural analysis of these interactions would bolster our understanding of the precise relationship between the signature effects of TM and Pgp-mediated drug binding and efflux [71].

In this study, a modulatory influence of TM on the responses of UWOV2 cells to commonly used anticancer drugs was noted by the significant decreases in EC_50 _of these drugs when used in combination with the antibiotic. The observed inhibition of VCR efflux from and its increased intracellular retention in UWOV2 cells following treatment with TM is in agreement with assertions that Pgp becomes inactive when its maturation, i.e., post-translational modification (N-glycosylation) is inhibited [25,28,30,99]. Hence, by blocking active efflux of the drug, TM may enhance chemosensitivity in terms of drug efficacy (potency) by increasing the net intracellular availability of the drug to wield its cytotoxic charge. Several studies suggest that the down-regulation of drug efflux may prove beneficial in overcoming drug resistance [29,98,100-103]. Lack of agreement on this posit may be explained by the fact that MDR is a multifactorial phenomenon in which the expression of alternative biochemical pathways related to defence and detoxification mechanisms, alterations in drug-target interactions and cellular responses to DNA damage are interlinked, yet phenotype-specific. By analogy, the molecular complexities in the systems studied and the diverse effects of TM at the cellular level may also account for conflicting observations.

This study has demonstrated that TM significantly increases the toxicity of various anticancer drugs in UWOV2 ovarian cystadenocarcinoma cells. The conformity of the data to the quantitative index of drug combination (I_x_) suggests that when TM is used conjointly with anticancer drugs, their efficacy is greatly enhanced through synergistic interaction, consistent with both the Loewe additivity and Bliss independence models [73,79,80]. The results have definite applications in research on refractory tumours, judging by the current gravity of the concept of synergy from the perspective of augmented drug potency and the promise it holds for drug discovery, development and optimization of adjuvant combinations [58,74]. The effects of TM in our experimental system were achieved through overall perturbation of the synthesis and function of proteins and glycoproteins. In this regard, Pgp, by virtue of its expression on UWOV2 cell surfaces and as one of the most likely causative molecules mediating ovarian tumour non-responsiveness, is not exempt from such targeting. Thus, the modulatory and inhibitory effects of TM on the dolichol-N-glycosylation pathway may be exploited *in vitro *as a useful strategy to interfere with the *de novo *formation of Pgp in order to render cells hypersensitive to anticancer agents. The relationship between drug synergism and mechanism is increasingly being recognized as an important consideration in multitherapeutic clinical rationales that would offer superior efficacy and lesser toxicity [58,74].

This study sought to address the broad mechanistic aspects underlying the observed synergistic responses of UWOV2 cells to TM-drug combinations. Further studies using immunohistochemical or flow-cytometry techniques are needed to profile the effect of TM on the expression levels of different drug efflux pumps in the UWOV2 cell line. The effect of TM described here may not be specific to ABC transporters, but rather universal. This opens new avenues to differentiate the TM-mediated effect between cancer cells and normal cells and, in particular, how such cellular sensitivities correlate with biomarkers for MDR [4]. The clinical application of TM, however, must await careful investigations relating to its *in vivo *toxicity.

## Abbreviations

MDR, Multidrug resistance; TM, tunicamycin; Pgp, P-glycoprotein; DXR, doxorubicin; EXR, epidoxorubicin/epirubicin; VCR, vincristine; CDDP, cis-diaminedichloroplatinum (II); AZD, azidopine; EC50, concentration that is 50% effective; CI, confidence interval; I_x_, drug interaction index; ER, endoplasmic reticulum; ERAD, ER-associated protein degradation; UPR, unfolded protein response; UPS, ubiquitin-proteasome system.

## Competing interests

The author(s) declare that they have no competing interests.

## Supplementary Material

Additional file 1 **Figure 1**. Effects of tunicamycin (TM) on protein synthesis (A) and glycoprotein synthesis (B) in UWOV2 ovarian carcinoma cells in culture. Protein synthesis was monitored by measuring the incorporation of [^35^S]methionine into cellular protein at various time intervals in the absence (control) or presence (TM-treated) of the antibiotic. Glycoprotein synthesis as a function of TM concentration was evaluated by determining the amount of [^3^H]glucosamine incorporated into cellular protein after 16h of exposure to the antibiotic. Data represent means ± SEM (n = 4). Two-tailed p values for the difference between control and TM-treated cells are presented within bars. Click here for file

Additional file 2 **Figure 2**. Effect of tunicamycin (5 μg/ml) on the incorporation of [^3^H]mannose by UWOV2 ovarian carcinoma cells in culture and the corresponding level of inhibition of mannosylglycoprotein synthesis. Values are means ± SEM (n = 4). Click here for file

Additional file 3 **Figure 3**. The effects of TM on drug cytotoxicity in UWOV2 ovarian carcinoma cells. Cells were seeded at a density of 3 × 10^3^ cells/well in octuplicate wells and allowed to attach and grow for 48–72h. Cells were were exposed to TM, drug, or to drug in combination with a fixed concentration of 5 μg/ml TM for a further 72h after which cell survival was determined by the MTT assay. A, DXR; B, EXR; C, VCR; D, CDDP. Data points are connected by non-linear regression lines of the sigmoidal dose-response relation. Values are means ± SEM for 3 experiments (n = 8 for each experiment). Click here for file

Additional file 4 **Table 1 (Must form part of the main document). Table 1**. Tunicamycin-induced sensitization of UWOV2 ovarian carcinoma cells to various antineoplastic drugs Click here for file

Additional file 5 **Figure 4**. Synergy analysis of the different antineoplastic drug and TM combinations in UWOV2 ovarian carcinoma cells. The median-effect function of Chou and Talalay, assuming mutual exclusivity, using the CombiTool (version 2.001) was applied to analyze both the Loewe additivity (A) and Bliss independence (B) reference models. A quantitative measure of drug interactions is provided by the interaction index of the isobologram equation (C). Plots show the different combination indices at various effect levels (fraction affected) for an experimental design in which the doses of the respective antineoplastic drugs were varied in the presence of a fixed dose of TM. The dashed line indicates the Loewe additivity hypothesis, i.e. interaction indices greater than 1 were antagonistic and those less than l were synergistic. Values are means for 3 experiments (n = 8 for each experiment). The coefficient of variation for each set of experiments was < 10%. Click here for file

Additional file 6 **Figure 5**. Time course of VCR uptake (A) and efflux (B) in human UWOV2 ovarian carcinoma cells. To assay VCR uptake and efflux, cells were pretreated for 16h with 5 μg/ml TM. Parallel controls were set up. Total cellular accumulation of [G-^3^H]VCR was determined at the end of each incubation period as described in Materials and methods. Efflux was measured by loading control and TM-pretreated cells with [G-^3^H]VCR for 60 min (0-time value for efflux) followed by washing preloaded cells three times with ice-cold PBS and subsequently incubating at 37°C in serum- and antibiotic-free medium for various time intervals. The absence or presence of TM was maintained throughout the post-incubation periods. Cells were harvested as described for uptake studies. Values are means ± SEM (n = 4) of 3 experiments. Student's two-tailed p values for the difference between control and TM-treated cells for the different time points are shown on top of panels. Click here for file

Additional file 7 **Figure 6**. Effects of tunicamycin on the retention of vincristine in drug-resistant UWOV2 human ovarian carcinoma cells at different time intervals after a 1-hr pre-loading period with the drug and subsequent exposure to drug-free medium. Values are means ± SEM (n = 4). Student's two-tailed p values for the difference between control and TM-treated cells are presented within bars. Click here for file

Additional file 8 **Figure 7**. Specific binding of [^14^C]DXR (A) and [^3^H]AZD (B) to cultured UWOV2 cells at 4°C for 60 min in the absence (control) or presence (TM-treated) of 5 μg/ml TM following an initial 16-h pre-incubation at 37°C with or without TM. Specific binding of DXR and AZD was calculated by subtracting non-specific binding data obtained in the presence of 100 μM each of unlabelled DXR and AZD, respectively, from total binding. Each point represents the mean ± SEM (n = 4). Upaired t-test results for the difference between control and TM-treated: p = 0.018 (10 nM ^14^C-DXR), p = 0.005 (20 nM ^14^C-DXR); p = 0.002 (40 -80 nM ^14^C-DXR), p ≤ 0.008 (10, 20, 40 and 80 nM ^3^H-AZD), p < 0.0001 (30 nM ^3^H-AZD), p = 0.012 (80 nM ^3^H-AZD, for difference between Equimolar DXR vs Equimolar DXR + TM). Click here for file
