# Supplementary material for: Combination of tunicamycin with anticancer drugs synergistically enhances their toxicity in multidrug-resistant human ovarian cystadenocarcinoma cells
Source: Cancer Cell Int. 2007 Apr 18;7:5. doi: 10.1186/1475-2867-7-5 (PMC1865531; doi:10.1186/1475-2867-7-5)
Supplement: Additional file 1 — Figure 1. Effects of tunicamycin (TM) on protein synthesis (A) and glycoprotein synthesis (B) in UWOV2 ovarian carcinoma cells in culture. Protein synthesis was monitored by measuring the incorporation of [35S]methionine into cellular protein at various time intervals in the absence (control) or presence (TM-treated) of the antibiotic. Glycoprotein synthesis as a function of TM concentration was evaluated by determining the amount of [3H]glucosamine incorporated into cellular protein after 16h of exposure to the antibiotic. Data represent means ± SEM (n = 4). Two-tailed p values for the difference between control and TM-treated cells are presented within bars. [file 1475-2867-7-5-S1.doc]

**Figure 1**

Effects of tunicamycin (TM) on protein synthesis (A) and glycoprotein synthesis (B) in UWOV2 ovarian carcinoma cells in culture. Protein synthesis was monitored by measuring the incorporation of [^35^S]methionine into cellular protein at various time intervals in the absence (control) or presence (TM-treated) of the antibiotic. Glycoprotein synthesis as a function of TM concentration was evaluated by determining the amount of [^3^H]glucosamine incorporated into cellular protein after 16h of exposure to the antibiotic. Data represent means ± SEM (n=4). Two-tailed p values for the difference between control and TM-treated cells are presented within bars.
