# Supplementary material for: Combination of tunicamycin with anticancer drugs synergistically enhances their toxicity in multidrug-resistant human ovarian cystadenocarcinoma cells
Source: Cancer Cell Int. 2007 Apr 18;7:5. doi: 10.1186/1475-2867-7-5 (PMC1865531; doi:10.1186/1475-2867-7-5)
Supplement: Additional file 2 — Figure 2. Effect of tunicamycin (5 μg/ml) on the incorporation of [3H]mannose by UWOV2 ovarian carcinoma cells in culture and the corresponding level of inhibition of mannosylglycoprotein synthesis. Values are means ± SEM (n = 4). [file 1475-2867-7-5-S2.doc]

**Figure 2**

Effect of tunicamycin (5 µg/ml) on the incorporation of [^3^H]mannose by UWOV2 ovarian carcinoma cells in culture and the corresponding level of inhibition of mannosylglycoprotein synthesis. Values are means ± SEM (n=4).
