# Supplementary material for: Combination of tunicamycin with anticancer drugs synergistically enhances their toxicity in multidrug-resistant human ovarian cystadenocarcinoma cells
Source: Cancer Cell Int. 2007 Apr 18;7:5. doi: 10.1186/1475-2867-7-5 (PMC1865531; doi:10.1186/1475-2867-7-5)
Supplement: Additional file 3 — Figure 3. The effects of TM on drug cytotoxicity in UWOV2 ovarian carcinoma cells. Cells were seeded at a density of 3 × 10^3^ cells/well in octuplicate wells and allowed to attach and grow for 48–72h. Cells were were exposed to TM, drug, or to drug in combination with a fixed concentration of 5 μg/ml TM for a further 72h after which cell survival was determined by the MTT assay. A, DXR; B, EXR; C, VCR; D, CDDP. Data points are connected by non-linear regression lines of the sigmoidal dose-response relation. Values are means ± SEM for 3 experiments (n = 8 for each experiment). [file 1475-2867-7-5-S3.doc]

**Figure 3**

The effects of TM on drug cytotoxicity in UWOV2 ovarian carcinoma cells. Cells were seeded at a density of 3 x 10^3^ cells/well in octuplicate wells and allowed to attach and grow for 48-72h. Cells were were exposed to TM, drug, or to drug in combination with a fixed concentration of 5 µg/ml TM for a further 72h after which cell survival was determined by the MTT assay. A, DXR; B, EXR; C, VCR; D, CDDP. Data points are connected by non-linear regression lines of the sigmoidal dose-response relation. Values are means ± SEM for 3 experiments (n=8 for each experiment).
