# Supplementary material for: Combination of tunicamycin with anticancer drugs synergistically enhances their toxicity in multidrug-resistant human ovarian cystadenocarcinoma cells
Source: Cancer Cell Int. 2007 Apr 18;7:5. doi: 10.1186/1475-2867-7-5 (PMC1865531; doi:10.1186/1475-2867-7-5)
Supplement: Additional file 4 — Table 1 (Must form part of the main document). Table 1. Tunicamycin-induced sensitization of UWOV2 ovarian carcinoma cells to various antineoplastic drugs [file 1475-2867-7-5-S4.doc]

**Table 1**

**Tunicamycin-induced sensitization of UWOV2 ovarian carcinoma cells to various antineoplastic drugs**

| **Drug** |  | **Alone^**^** | |  | **Combination with TM^**^** | |  | **Potency ratio^§^**  **(95 % CI; p^‡‡^)** |  | **Isobolar analysis of drug combinations^¶^** | | |
| --- | --- | --- | --- | --- | --- | --- | --- | --- | --- | --- | --- | --- |
|  |  | **EC_50_^*^** | **95 % CI^‡^** |  | **EC_50_^*^** | **95 % CI** |  |  |  | **Index (I_x_)^║,∫^** |  | **Interpretation** |
| **DXR** |  | 3.95 | 3.19 to 4.88 |  | 0.05 | 0.03 to 0.06 |  | 88 (62 to 126; p<0.001) |  | 0.15 ± 0.002 (n=9)**^†^** |  | Synergism |
| **EXR** |  | 2.87 | 0.76 to 10.80 |  | 0.03 | 0.02 to 0.04 |  | 102 (29 to 354; p<0.001) |  | 0.23 ± 0.002 (n=7) |  | Synergism |
| **VCR** |  | 23.2 | 9.11 to 59.07 |  | 4.60 | 0.10 to 208 |  | 5 (0.14 to 177; p=0.335) |  | 0.54 ± 0.003 (n=10) |  | Synergism |
| **CDDP** |  | 4.07 | 3.68 to 4.51 |  | 0.24 | 0.13 to 0.44 |  | 17 (10 to 31; p<0.001) |  | 0.33 ± 0.002 (n=9) |  | Synergism |

**^*^**Data (µg/ml) are best-fit values obtained from non-linear regression analysis of the sigmoidal dose-response relation for each drug alone or in combination with a fixed concentration of 5 µg/ml TM. **^‡^**CI, confidence interval; **^§^**The potency ratio and associated 95% CI were computed using GraphPad QuickCalcs ([www.graphpad.com](http://www.graphpad.com/)) according to the method of Fieller [82] by subtracting the log EC_50_ of drug in combination with TM from the log EC_50_ of drug alone and back-transformation (antilog) of data; ^¶^Analysis according to Chou and Talalay [81], using the CombiTool computer programme (version 2.001, www.imb-jena.de); I_x_, the drug interaction index [73,79,80], ^║^Values are geometric means ± **^†^**SEM (standard error of the mean) of multiple effect levels for each drug in the dose range 10^-4^ to 10^1^ µg/ml in the presence of a fixed concentration of 5 µg/ml TM; I_x_<1 ⇒ synergy; I_x_=1 ⇒ additivity and I_x_>1 ⇒ antagonism; **^‡‡^**Obtained from unpaired t-tests for the difference between log EC_50_ for drug alone and drug in combination with TM; **One-way ANOVA for the differences in log EC_50_ among treatment groups yielded p<0.001 in all cases, except for VCR alone vs VCR in combination with TM which showed marginal significance: p=0.061). ^∫^Student t-tests to evaluate significant differences in I_x_ compared to a null hypothesized I_x_ value of 1, yielded p values < 0.001 in all cases.
