# Supplementary material for: Combination of tunicamycin with anticancer drugs synergistically enhances their toxicity in multidrug-resistant human ovarian cystadenocarcinoma cells
Source: Cancer Cell Int. 2007 Apr 18;7:5. doi: 10.1186/1475-2867-7-5 (PMC1865531; doi:10.1186/1475-2867-7-5)
Supplement: Additional file 5 — Figure 4. Synergy analysis of the different antineoplastic drug and TM combinations in UWOV2 ovarian carcinoma cells. The median-effect function of Chou and Talalay, assuming mutual exclusivity, using the CombiTool (version 2.001) was applied to analyze both the Loewe additivity (A) and Bliss independence (B) reference models. A quantitative measure of drug interactions is provided by the interaction index of the isobologram equation (C). Plots show the different combination indices at various effect levels (fraction affected) for an experimental design in which the doses of the respective antineoplastic drugs were varied in the presence of a fixed dose of TM. The dashed line indicates the Loewe additivity hypothesis, i.e. interaction indices greater than 1 were antagonistic and those less than l were synergistic. Values are means for 3 experiments (n = 8 for each experiment). The coefficient of variation for each set of experiments was < 10%. [file 1475-2867-7-5-S5.doc]

**Figure 4**

Synergy analysis of the different antineoplastic drug and TM combinations in UWOV2 ovarian carcinoma cells. The median-effect function of Chou and Talalay, assuming mutual exclusivity, using the CombiTool (version 2.001) was applied to analyze both the Loewe additivity (A) and Bliss independence (B) reference models. A quantitative measure of drug interactions is provided by the interaction index of the isobologram equation (C). Plots show the different combination indices at various effect levels (fraction affected) for an experimental design in which the doses of the respective antineoplastic drugs were varied in the presence of a fixed dose of TM. The dashed line indicates the Loewe additivity hypothesis, i.e. interaction indices greater than 1 were antagonistic and those less than l were synergistic. Values are means for 3 experiments (n=8 for each experiment). The coefficient of variation for each set of experiments was <10%.
