# Supplementary material for: Combination of tunicamycin with anticancer drugs synergistically enhances their toxicity in multidrug-resistant human ovarian cystadenocarcinoma cells
Source: Cancer Cell Int. 2007 Apr 18;7:5. doi: 10.1186/1475-2867-7-5 (PMC1865531; doi:10.1186/1475-2867-7-5)
Supplement: Additional file 6 — Figure 5. Time course of VCR uptake (A) and efflux (B) in human UWOV2 ovarian carcinoma cells. To assay VCR uptake and efflux, cells were pretreated for 16h with 5 μg/ml TM. Parallel controls were set up. Total cellular accumulation of [G-3H]VCR was determined at the end of each incubation period as described in Materials and methods. Efflux was measured by loading control and TM-pretreated cells with [G-3H]VCR for 60 min (0-time value for efflux) followed by washing preloaded cells three times with ice-cold PBS and subsequently incubating at 37°C in serum- and antibiotic-free medium for various time intervals. The absence or presence of TM was maintained throughout the post-incubation periods. Cells were harvested as described for uptake studies. Values are means ± SEM (n = 4) of 3 experiments. Student's two-tailed p values for the difference between control and TM-treated cells for the different time points are shown on top of panels. [file 1475-2867-7-5-S6.doc]

**Figure 5**

Time course of VCR uptake (A) and efflux (B) in human UWOV2 ovarian carcinoma cells. To assay VCR uptake and efflux, cells were pretreated for 16h with 5 µg/ml TM. Parallel controls were set up. Total cellular accumulation of [G-^3^H]VCR was determined at the end of each incubation period as described in Materials and methods. Efflux was measured by loading control and TM-pretreated cells with [G-^3^H]VCR for 60 min (0-time value for efflux) followed by washing preloaded cells three times with ice-cold PBS and subsequently incubating at 37°C in serum- and antibiotic-free medium for various time intervals. The absence or presence of TM was maintained throughout the post-incubation periods. Cells were harvested as described for uptake studies. Values are means ± SEM (n=4) of 3 experiments. Student’s two-tailed p values for the difference between control and TM-treated cells for the different time points are shown on top of panels.
