# Supplementary material for: Combination of tunicamycin with anticancer drugs synergistically enhances their toxicity in multidrug-resistant human ovarian cystadenocarcinoma cells
Source: Cancer Cell Int. 2007 Apr 18;7:5. doi: 10.1186/1475-2867-7-5 (PMC1865531; doi:10.1186/1475-2867-7-5)
Supplement: Additional file 7 — Figure 6. Effects of tunicamycin on the retention of vincristine in drug-resistant UWOV2 human ovarian carcinoma cells at different time intervals after a 1-hr pre-loading period with the drug and subsequent exposure to drug-free medium. Values are means ± SEM (n = 4). Student's two-tailed p values for the difference between control and TM-treated cells are presented within bars. [file 1475-2867-7-5-S7.doc]

**Figure 6**

Effects of tunicamycin on the retention of vincristine in drug-resistant UWOV2 human ovarian carcinoma cells at different time intervals after a 1-hr pre-loading period with the drug and subsequent exposure to drug-free medium. Values are means ± SEM (n=4). Student's two-tailed p values for the difference between control and TM-treated cells are presented within bars.
