# Supplementary material for: Combination of tunicamycin with anticancer drugs synergistically enhances their toxicity in multidrug-resistant human ovarian cystadenocarcinoma cells
Source: Cancer Cell Int. 2007 Apr 18;7:5. doi: 10.1186/1475-2867-7-5 (PMC1865531; doi:10.1186/1475-2867-7-5)
Supplement: Additional file 8 — Figure 7. Specific binding of [14C]DXR (A) and [3H]AZD (B) to cultured UWOV2 cells at 4°C for 60 min in the absence (control) or presence (TM-treated) of 5 μg/ml TM following an initial 16-h pre-incubation at 37°C with or without TM. Specific binding of DXR and AZD was calculated by subtracting non-specific binding data obtained in the presence of 100 μM each of unlabelled DXR and AZD, respectively, from total binding. Each point represents the mean ± SEM (n = 4). Upaired t-test results for the difference between control and TM-treated: p = 0.018 (10 nM 14C-DXR), p = 0.005 (20 nM 14C-DXR); p = 0.002 (40 -80 nM 14C-DXR), p ≤ 0.008 (10, 20, 40 and 80 nM 3H-AZD), p < 0.0001 (30 nM 3H-AZD), p = 0.012 (80 nM 3H-AZD, for difference between Equimolar DXR vs Equimolar DXR + TM). [file 1475-2867-7-5-S8.doc]

**Figure 7**

Specific binding of [^14^C]DXR (A) and [^3^H]AZD (B) to cultured UWOV2 cells at 4°C for 60 min in the absence (control) or presence (TM-treated) of 5µg/ml TM following an initial 16-h pre-incubation at 37°C with or without TM. Specific binding of DXR and AZD was calculated by subtracting non-specific binding data obtained in the presence of 100 µM each of unlabelled DXR and AZD, respectively, from total binding. Each point represents the mean±SEM (n=4). Upaired t-test results for the difference between control and TM-treated: p=0.018 (10 nM ^14^C-DXR), p=0.005 (20 nM ^14^C-DXR); p=0.002 (40 -80 nM ^14^C-DXR), p≤0.008 (10, 20, 40 and 80 nM ^3^H-AZD), p<0.0001 (30 nM ^3^H-AZD), p=0.012 (80 nM ^3^H-AZD, for difference between Equimolar DXR vs Equimolar DXR + TM).
